# Isolation and Characterization of NP-POL Nonapeptide for Possible Therapeutic Use in Parkinson's Disease

**DOI:** 10.1155/2018/3760124

**Published:** 2018-07-18

**Authors:** Marta Lemieszewska, Antoni Polanowski, Tadeusz Wilusz, Agata Sokołowska, Aleksandra Zambrowicz, Katarzyna Mikołajewicz, Józefa Macała, Joanna Rymaszewska, Agnieszka Zabłocka

**Affiliations:** ^1^Laboratory of Signal Transduction Molecules, Hirszfeld Institute of Immunology and Experimental Therapy, Polish Academy of Sciences, Rudolfa Weigla 12, 53-114 Wrocław, Poland; ^2^Department of Psychiatry, Wrocław Medical University, Pasteura 10, 50-367 Wrocław, Poland; ^3^Department of Animal Products Technology and Quality Management, Faculty of Biotechnology and Food Sciences, Wrocław University of Environmental and Life Sciences, Chełmońskiego 37, 51-630 Wrocław, Poland; ^4^Faculty of Biotechnology, University of Wrocław, Joliot-Curie 14a, 50-383 Wrocław, Poland; ^5^Laboratory of Reproductive Immunology, Hirszfeld Institute of Immunology and Experimental Therapy, Polish Academy of Sciences, Rudolfa Weigla 12, 53-114 Wrocław, Poland

## Abstract

Colostrum and milk are the initial mammalian nourishment and rich reservoir of essential nutrients for newborn development. Bioactive peptides isolated from natural sources, such as colostrum, serve as endogenous regulators and can be used as alternative therapeutic agents in the treatment of neurodegenerative diseases. One example is the previously unknown NP-POL nonapeptide isolated from Colostrinin. In the present study, we investigated a method of NP-POL nonapeptide isolation using Bio-Gel P2 molecular sieve chromatography. We showed the protective effect of NP-POL on 6-hydroxydopamine- (6-OHDA-) induced neurotoxicity using rat adrenal pheochromocytoma (PC12 Tet On) cells. Treatment of PC12 cells with NP-POL nonapeptide reduced 6-OHDA-induced apoptosis and caused transient phosphorylation of extracellular signal-regulated kinases (ERK 1/2), which were shown to promote cell survival. NP-POL nonapeptide also protected neuronal cells against oxidative injury induced by 6-OHDA. These results showed a potential use of NP-POL in the therapy of Parkinson's disease.

## 1. Introduction

Parkinson's disease (PD) is considered the second most common neurodegenerative disease after Alzheimer's disease, involving 0.3% of industrialized country populations, with a prevalence rising with age from 1% in people over 60 years of age to 4% in those over 80 [1, 2]. PD results from the progressive loss of dopaminergic neurons in the parts of the brain that control muscle movement—the basal ganglia and the extrapyramidal area. Pathological indicators of PD are cytoplasmic inclusions—Lewy bodies and massive atrophy of dopaminergic neurons in substantia nigra pars compacta. Clinically, PD is characterized by motor symptoms (such as bradykinesia, hypokinesia, cogwheel rigidity, resting tremor, and postural instability), sleep disorders, hyposomia, anxiety, and depression [3]. Although the etiology of Parkinson's disease is still not completely clear, some causes have been found, including neuroinflammation, genetic mutation of genes, and mitochondrial and proteasomal dysfunction, as well as *α*-synuclein aggregation [1, 2].

Several studies have reported that the overproduction of free oxygen radicals and an impaired antioxidative defense system are initial steps in PD [4, 5]. Oxidative stress is a product of an imbalance between oxidative and antioxidative systems in cells, generating increased levels of free oxygen and nitrogen radicals, leading to impairment in proteins, lipids, and DNA, as well as mitochondrial dysfunction [6, 7]. High levels of redox active metals, decreased activity of antioxidant enzymes, and reduced level of glutathione play a pivotal role in the etiology of PD [8, 9].

Reactive oxygen species (ROS) are also generated in dopaminergic neurons during enzymatic degradation of dopamine by monoamine oxidase, as well as during nonenzymatic dopamine autoxidation to neuromelanin [10, 11]. 6-Hydroxydopamine (6-OHDA) detected in both rat and human brains after long-term L-3,4-dihydroxyphenylalanine (L-DOPA) administration has also been proposed as a neurotoxin in the pathogenesis of PD [12, 13]. 6-OHDA acts via inducing ROS overproduction and energy depletion [13–15]. The toxic effect of 6-OHDA results from the overproduction of ROS through three pathways: extracellular autooxidation, intracellular metabolism by monoamine oxidase, and direct inhibition of the mitochondrial respiratory chain. The increase in the level of ROS by 6-OHDA leads to a decrease in cellular antioxidant enzymes and, subsequently, neuronal apoptosis [16].

The treatment of PD has not changed substantially in the past 30 years, with the key role of dopamine replacement therapy, including L-3,4-dihydroxyphenylalanine (L-DOPA) and dopamine agonists, supported by the use of peripheral decarboxylase inhibitors, catechol-O-methyl transferase inhibitors, and monoamine oxidase-B (MAO-B) inhibitors [17]. The current treatments do not prevent the continuing loss of dopamine neurons, and eventually treatment-related side effects result in severe disability.

One viable alternative to the drugs used in treating PD can be natural products—nutraceuticals or functional foods—commonly used for preventing or attenuating the process of aging. The strong therapeutic potential of bioactive compounds obtained from natural products in age-related disorders such as Parkinson's disease is associated with their multidirectional action. They are more available and considerably safer to use and can promote prosurvival signals and act as antioxidants [18, 19]. These substances can act as agonists for dopaminergic neurons, improve cognitive function, promote mitochondrial function, inhibit ROS generation, and also possess immunomodulatory activity [20, 21]. An example of such a compound is the previously unknown nonapeptide NP-POL isolated as a component of the proline-rich polypeptide complex (PRP, also known as Colostrinin).

PRP is a complex of low-molecular weight peptides ranging from 500 Da to 3000 Da, first isolated from ovine colostrum, and also present in human, bovine, and caprine colostrum [22]. It is one of the many important constituents of colostrum, the first mammalian nourishment, which may stimulate the neonate immune system and play a regulatory role in newborn development, next to immunoglobulins, cytokines, and lymphokines. It is active both *in vivo* and *in vitro* and is not cytotoxic even at 1.25 g/kg body weight.

Because of its multicomponent character, PRP shows pleiotropic activity. It has immunoregulatory properties, regulating both humoral and cellular immune responses. It modulates the innate immune response, including phagocytosis and the balance between oxidants and antioxidants, thus regulating redox-sensitive cellular signaling [23–25]. Additionally, PRP can affect learning, memory, and lifespan and possesses neuroprotective activity [23, 26, 27]. The activity of PRP suggests a potential therapeutic use in the case of diseases associated with changes in innate immunity, for example, Alzheimer's disease [23]. It has also been suggested that PRP has potential in treating other neurodegenerative diseases, such as multiple sclerosis, Parkinson's disease, and amyotrophic lateral sclerosis.

The present study shows a method of isolation and purification of a previously unknown PRP constituent, NP-POL nonapeptide. In addition, we used the PC12 Tet On cell line to investigate the protective effect of NP-POL in 6-OHDA-induced oxidative stress. Our study provides new evidence that NP-POL may protect PC12 cells against 6-OHDA cytotoxicity through a neuroprotective and antioxidant activity. Our results indicate a potential use of NP-POL in the therapy of Parkinson's disease.

## 2. Materials and Methods

### 2.1. Reagents

High-glucose Dulbecco's modified Eagle's medium (DMEM) and phosphate-buffered saline (pH 7.4) (PBS) were sourced from the Laboratory of General Chemistry of the Institute of Immunology and Experimental Therapy, PAS (Poland). L-glutamine, antibiotics (penicillin/streptomycin mixture), donor horse serum, and fetal bovine serum (FBS) were produced by BioWest (Nuaille, France). Stabilized hydrogen peroxide 30%, 2,7-dichlorofluorescein diacetate (DCFH), 1,1-diphenyl-2-picrylhydrazyl (DPPH), ferrozine, Trolox, 3-(4,5-dimethylthiazol-2-yl)-2-5-diphenyltetrazolium bromide (MTT), 2′,7′-dichlorofluorescein diacetate (DCFH-DA), and Tween 20 were from Sigma (St. Louis, MO, USA). Reagents for SDS-PAGE were from Bio-Rad (California, USA). 2.5S NGF (from mouse submaxillary glands) and BDNF Emax ImmunoAssay System were from Promega (Madison, USA). Page Ruler™ Plus Prestained Protein Ladder (10 kDa–250 kDa) was obtained from Thermo Scientific (Waltham, MA, USA). 6-Hydroxydopamine (6-OHDA) was provided by Tocris Bioscience (Bristol, UK). Rabbit anti-ERK/anti-phosphoERK monoclonal antibodies and alkaline phosphatase-conjugated anti-rabbit IgG antibodies were from Cell Signaling Technology (MA, USA). 5-Bromo-4-chloro-3-indolyl phosphate disodium salt (BCIP) and nitroblue tetrazolium (NBT) were from Carl Roth GmbH (Karlsruhe, Germany).

Synthetic NP-POL peptide used to determine biological activity was obtained by chemical synthesis at Lipopharm (Gdańsk, Poland).

### 2.2. Isolation of NP-POL Nonapeptide from Colostrinin

#### 2.2.1. Isolation of Colostrinin

Sheep and bovine colostrum were obtained from sheep farms in Szczenyrz and Grywałd and the farm of the Wrocław University of Environmental and Life Sciences, Poland, respectively. Colostrum was collected from sheep and cow up to 24 hours after delivery.

**Figure 1:**
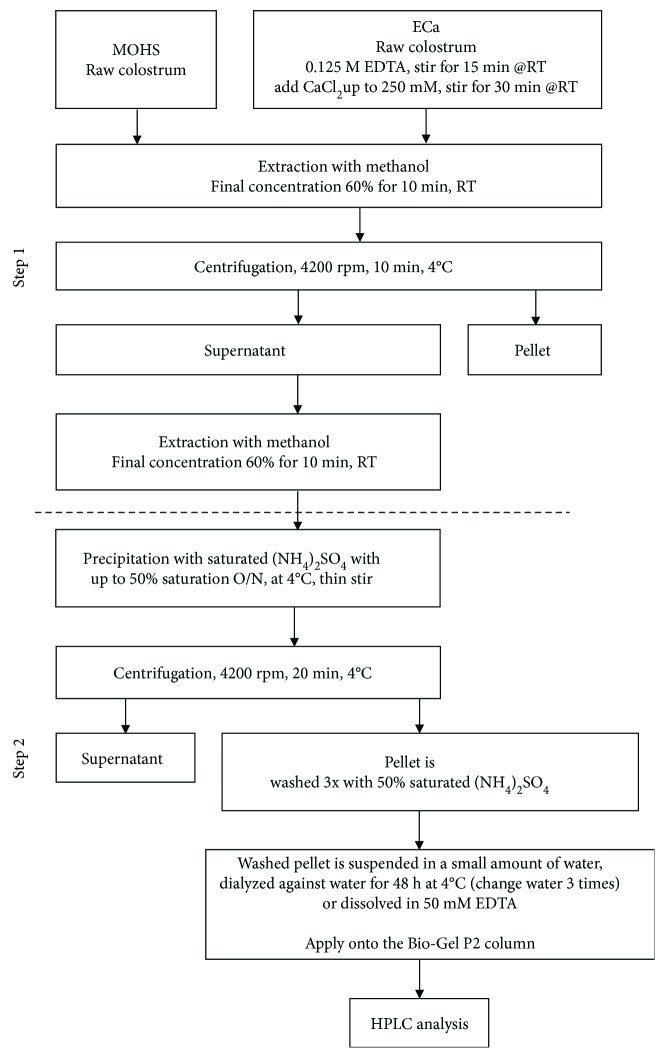
Diagram of the NP-POL peptide isolation process.

Colostrinin (CLN) was separated from colostrum using the classical method according to Janusz et al. [22]. Also, a two-step purification method based on the alcohol/salt extraction/precipitation procedure according to Kruzel et al. [28] was used. This method involves the extraction of peptides with 60% methanol directly from raw colostrum (MOHS sample) or after conditioning with EDTA/CaCl_2_ (ECa sample) and precipitation with 50% ammonium sulfate (Figure 1). A consistent pool of essentially IgG-free polypeptides is obtained at a high yield with these protocols.

#### 2.2.2. Separation of NP-POL Nonapeptide from CLN, MOHS, and ECa Samples

100 mg of Colostrinin or 10 mg of MOHS or 100 mg of ECa sample were dissolved in 5 ml, 1 ml, and 5 ml of 50 mM EDTA, respectively, and applied onto the Bio-Gel P2 molecular sieve beads equilibrated with the same solution. One ml fractions are collected and the protein profile plotted upon the protein concentration measured in each fraction.

#### 2.2.3. RP-HPLC

RP-HPLC analysis was carried out on a Nucleosil 100 C-18 column (particle size 10 *μ*m, 250 mm × 8 mm). The peptides of peak “c” eluted from Bio-Gel P2 were dissolved in 0.1% TFA and applied to a column equilibrated with 20% ACN in 0.1% TFA. The proteins were eluted with a linear gradient of ACN from 20% to 100% in 0.1% TFA (*v*/*v*) at a flow rate of 1 ml/min in 60 min and detected at 220 nm.

#### 2.2.4. SDS-PAGE

SDS/polyacrylamide slab gels were prepared under reducing conditions according to Schägger and von Jagow [29]. The gel slabs were cast 24 h ahead of electrophoresis and stained with Coomassie Brilliant Blue R-250 for proteins.

#### 2.2.5. Amino Acid Sequence Analysis

The amino acid sequence of the peptide separated from fraction 2 obtained after RP-HPLC analysis was made to undergo the Edman degradation method [30].

### 2.3. Cell Cultures

Whole blood samples from healthy donors were kindly provided by the Station of Blood Donation, 4th Military Hospital, Wrocław, Poland. Samples were collected into syringes containing 10 U/ml of heparin. Within 2 h of collection, the blood was diluted 10-fold with RPMI 1640 medium supplemented with 100 units/ml penicillin, 100 mg/ml streptomycin, and 0.5 mg/ml L-glutamine. Whole blood samples were used for the determination of cytokine level.

PC12 Tet On (ATCC) rat pheochromocytoma cells used as a model of neuronal cells were kindly provided by Professor Janusz Matuszyk (Institute of Immunology and Experimental Therapy, PAS, Wrocław). The cells were maintained under 5% CO_2_/95% humidified air at 37°C in Dulbecco modified Eagle's medium (DMEM), supplemented with 5% horse serum and 10% fetal bovine serum, antibiotics (penicillin and streptomycin), and 2 mM L-glutamine. The culture medium was changed once every three days.

### 2.4. Cytokine Induction and Determination

Cytokine secretion was induced according to the method described by Inglot et al. [31]. Blood samples were collected in syringes containing sodium heparin. Within 1 h of collection, the blood was diluted 10-fold with RPMI 1640 medium supplemented with penicillin/streptomycin and L-glutamine. One ml portions of the cell suspension were distributed in duplicate into 48-well flat-bottomed tissue culture plates. NP-POL at doses of 1 *μ*g–100 *μ*g was added to 100 *μ*l of RPMI 1640. As a reference, positive inducers were used: 2 *μ*g/ml of leucoagglutinin (PHA) and 2 *μ*g/ml of lipopolysaccharide (LPS). Control wells containing the nontreated blood cell samples were used to measure the spontaneous production of cytokines (negative control). The plates were incubated for 22 h at 37°C in a 5% CO_2_ atmosphere. After incubation, the supernatants were collected and used for the determination of cytokines. IL-1*β*, TNF-*α*, IL-6, and IL-10 were determined by an enzyme-linked immunosorbent assay using human IL-1*β*, TNF*α*, IL-6, and IL-10 ELISA Max™ Deluxe Kit (BioLegend, San Diego, CA) according to the procedure recommended by the manufacturer.

### 2.5. Determination of Cell Viability

Cell viability was determined using the MTT colorimetric assay [32]. PC12 cells were seeded onto poly-L-lysine-coated 96-well plates (1 × 10^4^/well) and next incubated for 24 h with inducers: NP-POL (1 *μ*g/ml–150 *μ*g/ml) or toxin 6-OHDA (1 *μ*M–200 *μ*M). To measure the neuroprotective effect, NP-POL nonapeptide was applied simultaneously with 6-OHDA (150 *μ*M) or preincubated for 1 h and then exposed to 150 *μ*M 6-OHDA for 24 h. After cell treatment, the supernatant was removed and the cells were incubated with MTT (5 mg/ml) for 4 h at 37°C. The formazan crystals were dissolved by adding 100 *μ*l of DMSO and vigorously shaking to complete resolving. The absorbance was measured by an EnSpire™ 2300 microplate reader (PerkinElmer, Massachusetts, USA) at 570 nm. Cell viability was expressed as a percentage of control.

### 2.6. Determination of Antioxidant Activity as the Ability to Scavenge DPPH Free Radicals

The antioxidant activity of NP-POL nonapeptide was assessed on the basis of the radical scavenging effect of stable 1,1-diphenyl-2-picrylhydrazyl free radical activity according to Yen and Chen [33], with minor modifications. The tested samples were dissolved in water to a final volume of 1 ml and mixed with 1 ml of ethanol (98%). The reaction was started by adding 0.5 ml of 0.3 mM DPPH in ethanol. The mixtures were left for 30 minutes at room temperature, and the absorbance of the resulting solutions was measured at 517 nm. For calibration, aqueous solutions of known Trolox concentrations ranging from 2 *μ*g to 20 *μ*g (able to scavenge 500 *μ*l of 0.3 mM DPPH radical solution) were used. Radical scavenging activity of the peptide was expressed as *μ*M Trolox_eq_.

### 2.7. FRAP Method

The FRAP method (ferric-reducing antioxidant power) was used to determine the antioxidative capacity of NP-POL according to Benzie and Strain [34]. 3 ml of FRAP working solution (300 mM acetate buffer pH 3.6; 10 mM 2,4,6-tripyridyl-s-triazine; and 20 mM FeCl_3_ × 6H_2_O (10 : 1 : 1 *v*/*v*)) was mixed with 1 ml of sample. After 10 min of reaction, the absorbance was measured at *λ* = 593 nm. An aqueous solution of known Fe(II) concentrations was used for calibration (in the range from 100 *μ*g to 1000 *μ*g). Results were expressed as *μ*g Fe^2+^.

### 2.8. Determination of Fe(II) Ion Chelation

Chelation of iron ions by the NP-POL peptide was estimated by the method of Xu et al. [35] with some modifications. A 250 *μ*l sample was mixed with 1250 *μ*l of H_2_O and 110 *μ*l of 1 mM FeCl_2_. After 2 min, 1 ml of 500 *μ*M ferrozine aqueous solution was added and the mixture was allowed to react for 10 minutes. The absorbance of the ferrous iron-ferrozine complex was measured spectrophotometrically at 562 nm. A known concentration of FeCl_2_ (0 *μ*g–20 *μ*g) was used to generate a standard curve, and the ability to chelate iron ions was expressed as *μ*g Fe^2+^.

### 2.9. Determination of Intracellular ROS Level

Intracellular ROS was analyzed using a 2′,7′-dichlorofluorescein diacetate (DCFH-DA) fluorescence assay. PC12 cells were plated onto 96-well poly-L-lysine-coated plates in DMEM culture medium 24 h before experiments. The NP-POL peptide was applied to the PC12 cells at 1 *μ*g/ml, 10 *μ*g/ml, and 100 *μ*g/ml simultaneously with 6-OHDA (150 *μ*M), 1 h before exposure to 6-OHDA, or without exposure to 6-OHDA. 24 hours later, the medium was removed, and the cells were washed with DMEM medium without FBS and then incubated with 25 *μ*M DCFH-DA for 40 min at 37°C. The fluorescence intensity was measured every 30 minutes at 485 nm (excitation) and 527 nm (emission) wavelengths on a microplate reader (PerkinElmer, Massachusetts, USA). Changes in absorbance were expressed as relative fluorescence units (RFU)/min. Data are presented as a percentage of control.

### 2.10. Determination of Protein Content

Protein concentration in the analyzed samples was determined by a bicinchoninic acid kit according to the manufacturer's suggestions.

### 2.11. Western Blot Analysis

PC12 cells (1×10^6^ cells/ml) were seeded onto poly-L-lysine-coated 6-well culture plates and incubated with NP-POL (100 *μ*g/ml), 6-OHDA (150 *μ*M), or NP-POL and 6-OHDA applied simultaneously, for 5 min, 10 min, 30 min, and 60 min at 37°C. Next, the cells were lysed by RIPA buffer (150 mM NaCl, 50 mM Tris-HCl pH 7.5, 5 mM EDTA, 1% Triton X-100, 0.1% SDS, and 0.5% deoxycholate) supplemented with a protease and phosphatase inhibitor cocktail (Roche), 1 mM NaF, and 2 mM Na_3_VO_4_ for 30 min in ice. Lysates were centrifuged at 14,000*g* for 10 min (4°C), and the protein content was measured using a BCA kit. 50 *μ*g of protein samples was separated on 12% sodium dodecyl sulfate- (SDS-) polyacrylamide gel and transferred to a nitrocellulose membrane. The membrane was blocked (Tris-HCl buffer, pH 7.0, 5% Tween 20 (TBS-T), and 5% nonfat dried milk) for 1 h at room temperature and then probed overnight at 4°C with primary antibodies anti-ERK 1/2 and anti-phospho-ERK 1/2 diluted 1 : 1000 in TBST with 5% BSA, and for 1 h at room temperature using secondary antibodies conjugated with alkaline phosphatase (1 : 10,000 in TBST with 5% BSA) according to the standard procedure. Immunocomplexes were visualized using a NBT/BCIP substrate and analyzed in a ChemiDoc MP Imaging System.

### 2.12. Analysis of Neurite Outgrowth (a) and Protection before 6-OHDA (b)

- PC12 cells (1×10^4^ cells/well) were plated onto poly-L-lysine-coated chamber slides (Nunc) and cultured in Opti-MEM-reduced serum medium (Gibco) at 37°C and 5% CO_2_ for 2 h. NP-POL (1 *μ*g/ml, 10 *μ*g/ml, and 100 *μ*g/ml) was added to the cells as a potential inducer of neuritogenesis. NGF (0.1 *μ*g/ml) was used as a positive control, while untreated PC12 cells were used as a negative control. PC12 cells were maintained at 37°C in 95% humidified atmosphere/5% CO_2_ for 3–6 days in the presence of the tested substances. Cells were observed by phase-contrast microscopy, and the number of neurite-positive cells was counted.
- PC12 cells (1 × 10^4^/well) were plated onto poly-L-lysine-coated chambered slides and cultured in Opti-MEM-reduced serum medium at 37°C and 5% CO_2_ for 2 h. When cells were settled, 0.1 *μ*g/ml of NGF was added into each well. After 96 h, differentiated PC12 cells were preincubated with NP-POL (1 *μ*g/ml–100 *μ*g/ml) for 1 h and then exposed to toxic 6-OHDA (150 *μ*M) for 24 h. NGF-treated cells were used as a positive control, and 6-OHDA-treated cells were used as negative control. Images of the treated cells were captured with a digital camera. The number of neurite-bearing cells was used to evaluate neurite outgrowth and retraction in response to treatment.

### 2.13. Statistical Analysis

Each experimental procedure was performed in at least three independent cell preparations with two replicates each. One-way ANOVA followed by Dunnett's multiple comparison test was used to compare control and treated groups with *p* < 0.05 considered statistically significant.

## 3. Results and Discussion

Colostrum and milk are the initial mammalian nourishment. They are the richest reservoir of important nutrients in newborn development. They contain protective and supporting factors, such as immunoglobulins, cytokines, and also lymphokines and peptides which may stimulate the neonate immune system and play a regulatory role [36, 37]. One of them is a proline-rich polypeptide complex (PRP) from ovine colostrum, also known as Colostrinin (CLN). PRP was first discovered over 30 years ago as an IgG_2_-PRP complex and was subsequently found in human, bovine, and caprine colostra [22]. The immunomodulatory activity of PRP suggests a therapeutic use in the case of diseases in which changes in innate immunity play a role, including neurodegenerative disorders [23]. The current study presents a method of isolation of a previously unknown NP-POL peptide from PRP and shows its potential biological role in the regulation of cellular mechanisms regulating the survival of nerve cells treated with neurotoxic 6-hydroxydopamine and its potential use as a pharmacological preparation in the treatment of Parkinson's disease.

### 3.1. NP-POL Peptide Isolation and Identification

**Figure 2:**
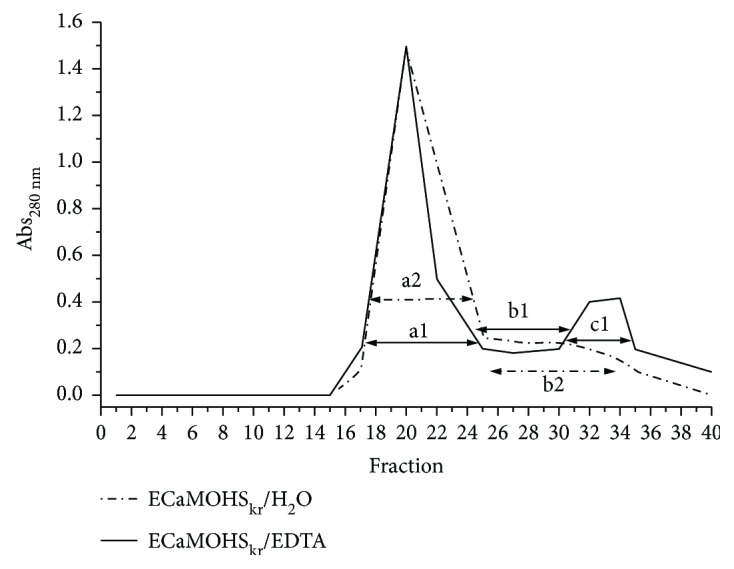
Separation of the NP-POL peptide from Colostrinin by Bio-Gel P2 molecular sieve beads.

PRP was isolated from colostrum using the method described by Janusz et al. [22]. A selective extraction with 60% methanol in a one-step protocol produced a PRP-rich solution, which after ammonium sulfate precipitation gave a final preparation, designated MOHS. Treatment of the colostrum with EDTA to dissolve casein micelles prior to alcohol extraction increased the yield of PRP several times [28]. The employment of EDTA allowed discovery of a previously unknown nonapeptide named NP-POL, which in the presence of a chelate was released from the complex (Figure 1). The nonapeptide precipitation obtained from 100 mg of an ECa sample led to the highest yield. The elution profile obtained after the separation of the ECa sample on Bio-Gel P2 in molecular sieve chromatography was characterized by the presence of a high peak of “a1” and a much lower peak of “c1” (Figure 2). The fraction “c1” was then fractionated by RP-HPLC. The obtained chromatogram showing the changes in absorbance at *λ* = 220 nm over time confirmed the presence in the “c1” peak of peptide fractions differing in hydrophobic/hydrophilic properties, released from C-18 by acetonitrile at a concentration of 15%–60% (see supplementary data available here). Electrophoretic analysis revealed the presence of peptides of MW about 1 kDa in fraction 2. Amino acid sequence analysis of subfraction “c1” revealed the presence of a nonapeptide with the Arg-Pro-Lys-His-Pro-Ile-Lys-His-Gln sequence. Screening of homologous proteins in the UniProt protein sequence database using the Fasta 3 program showed that the newly discovered NP-POL nonapeptide showed 100% homology to the 16- to 24-amino acid fragment of the sheep and beef alpha s1 casein precursor and 88.88% identity with alpha-s casein (see supplementary data).

### 3.2. Immunoregulatory Activity of NP-POL Nonapeptide

It has been previously shown that PRP modulates cytokine production by human whole blood cell cultures. In addition, PRP inhibits the production of nitric oxide (NO) induced by proinflammatory agents both *in vivo* and *in vitro* [23]. Cytokines are the proteins which stimulate or inhibit the activation, proliferation, and differentiation of various target cells upon antigen activation. They also participate in regulation of inflammation and immunity and are crucial for protection against infection and injury. In turn, NO is an important agent functioning as an effector molecule in biological signaling, connected with regulation of immune responses, cell differentiation, and apoptosis [4].

In the present study, we determined the cytokine- and NO-inducing activity of the previously unknown PRP complex constituent—NP-POL nonapeptide. Human whole blood cultures used as an experimental model ex vivo mimic the natural environment of immunocompetent cells and preserve the various intercellular communications between the different blood cell populations. In our study, we determined the levels of two types of cytokines: IL-1*β* and TNF-*α*, secreted by Th1 cells involved in cellular immunity, and IL-6 and IL-10 secreted by Th2 cells, participating in the humoral immune response. Additionally, murine bone marrow-derived macrophages (BMDM) were used as a model to determine the effect of NP-POL on NO production. The results obtained showed that the NP-POL peptide was not a cytokine or NO inducer and also did not inhibit production of nitric oxide (NO) induced by LPS (data not shown).

### 3.3. Neuroprotective Effect of NP-POL Nonapeptide

Parkinson's disease is one of the most common neurodegenerative movement disorders, caused by a selective loss of dopaminergic neuronal cells in the midbrain region substantia nigra pars compacta [1, 2]. One of the many causes of PD is the accumulation of free radicals and oxidative stress products which lead to selective neuronal loss [4]. Our work was designed to ascertain whether the newly discovered PRP component NP-POL nonapeptide has a potential neuroprotective and antiapoptotic capacity. We mainly focused on its activity against the neurotoxicity of 6-hydroxydopamine on neuronal PC12 cells and its potential antioxidant properties. 6-OHDA, which is a toxic dopamine analog, has been detected in both rat and human brains after long-term L-DOPA administration. It is a redox active neurotoxin which is commonly used to produce a Parkinsonian pattern of neuronal loss [12, 14].

#### 3.3.1. NP-POL Protects PC12 Cells from 6-OHDA-Induced Toxicity

**Figure 3:**
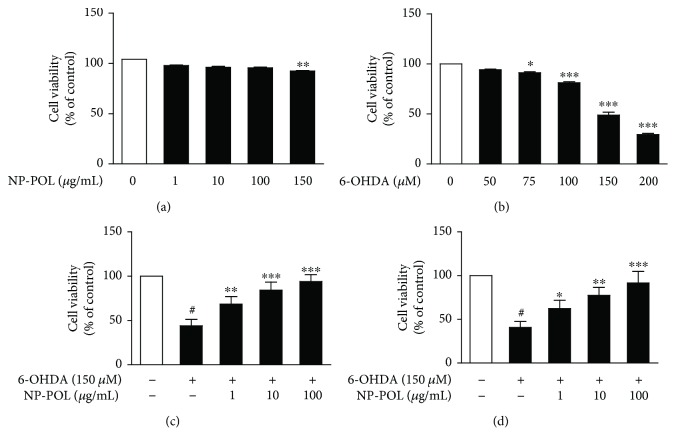
The effects of NP-POL on 6-OHDA-induced cytotoxicity of PC12 cells. PC12 cells (1 × 10^4^/well) were incubated for 24 h with inducers: NP-POL (1 *μ*g/ml–150 *μ*g/ml) (a) or toxin 6-OHDA (1 *μ*M–200 *μ*M) (b). To measure the neuroprotective effect of NP-POL, the nonapeptide was preincubated with cells for 1 h before application of 150 *μ*M 6-OHDA (c) or was applied simultaneously with 6-OHDA (150 *μ*M) (d). Cell viability was measured by the MTT assay. Data are presented as mean ± SD (*n* = 3). Results were considered significant at ^#^ *p* < 0.001 versus untreated cells and at ^∗^ *p* < 0.05, ^∗∗^ *p* < 0.01, and ^∗∗∗^ *p* < 0.001 versus 6-OHDA.

Firstly, the cytotoxic effect of NP-POL on PC12 Tet On cells was determined. Our results indicated that NP-POL at doses ranging from 1 *μ*g/ml to 150 *μ*g/ml neither displayed cytotoxicity nor showed any adverse effect on PC12 cell viability at higher concentrations (Figure 3(a)). Then, we investigated the protective effect of NP-POL against 6-OHDA toxicity. PC12 cell viability was reduced by 6-OHDA treatment in a dose-dependent manner. However, 150 *μ*M of 6-OHDA decreased PC12 cell viability by about 50%, and this dose was used to measure the neuroprotective activity of NP-POL (Figure 3(b)). It was shown that both 1 h pretreatment (Figure 3(c)) and also simultaneous application (Figure 3(d)) of NP-POL with 6-OHDA significantly increased cell survival in a dose-dependent manner. As was shown previously, PRP possesses neuroprotective activity against toxic amyloid *β*_42_, which may suggest a potential antiapoptotic activity [23]. The present work suggests that the NP-POL peptide could effectively protect PC12 cells from 6-OHDA, a dopaminergic neuron-damaging toxin.

#### 3.3.2. NP-POL Protects Differentiated PC12 Cells from 6-OHDA Toxicity

**Figure 4:**
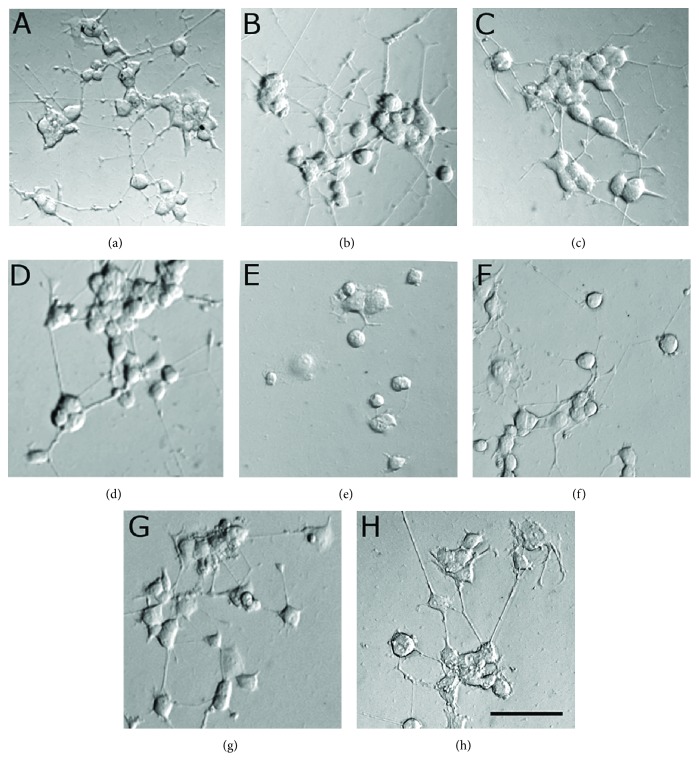
The neuroprotective effect of the NP-POL peptide on NGF-differentiated PC12 cell viability treated with 6-OHDA neurotoxin. (a) Control, (b) NP-POL 1 *μ*g/ml, (c) NP-POL 10 *μ*g/ml, (d) NP-POL 100 *μ*g/ml, (e) 6-OHDA 160 *μ*M, (f) NP-POL 1 *μ*g/ml + 6-OHDA 160 *μ*M, (g) NP-POL 10 *μ*g/ml + 6-OHDA 160 *μ*M, and (h) NP-POL 100 *μ*g/ml + 6-OHDA 160 *μ*M. Scale bar = 50 *μ*M.

The neuroprotective effect of NP-POL against 6-OHDA toxicity was also determined on an NGF-differentiated PC12 cell model. Firstly, we showed that undifferentiated PC12 cells cultivated with NP-POL for 5 days did not produce neurites (data not shown). Next, PC12 cells were differentiated in the presence of NGF for 5 days and then treated with NP-POL alone, neurotoxin 6-OHDA alone, or with NP-POL applied to the PC12 cells 1 h before the neurotoxin. As shown in Figures 4(b)–4(d), NP-POL did not show any toxic effect on the PC12 cells. When the cells were treated with 6-OHDA, the cell number was reduced, the cells began to swell, the neurites started to retract, the network collapsed, and cell debris appeared (Figure 4(e)). To investigate whether NP-POL had protective activity against 6-OHDA-induced neurite damage, the cells were preincubated for 1 h with NP-POL before 6-OHDA application (Figures 4(f)–4(h)). Compared with the cells exposed to 6-OHDA alone, 1 *μ*g/ml and 10 *μ*g/ml of NP-POL exhibited a weak protective effect (Figures 4(f) and 4(g). However, 100 *μ*g/ml of NP-POL peptide effectively increased cell viability, with significant protection of neurites and reduction of cell debris (Figure 4(h)).

#### 3.3.3. NP-POL Peptide Attenuates 6-OHDA-Induced ROS Generation

Overproduction of ROS and an impaired antioxidative defense system are some of the initial steps in PD pathology [4]. The toxic effect of 6-OHDA, used here to create experimental models of PD, can be linked to the overproduction of ROS in PC12 cells, which may increase the expression of redox-sensitive transcription factors responsible for oxidative and inflammatory reactions in PD and also leads to increased levels of toxic products of proteins and lipid oxidation, and also leads to severe mitochondrial dysfunction and neuronal apoptosis [14, 38].

**Figure 5:**
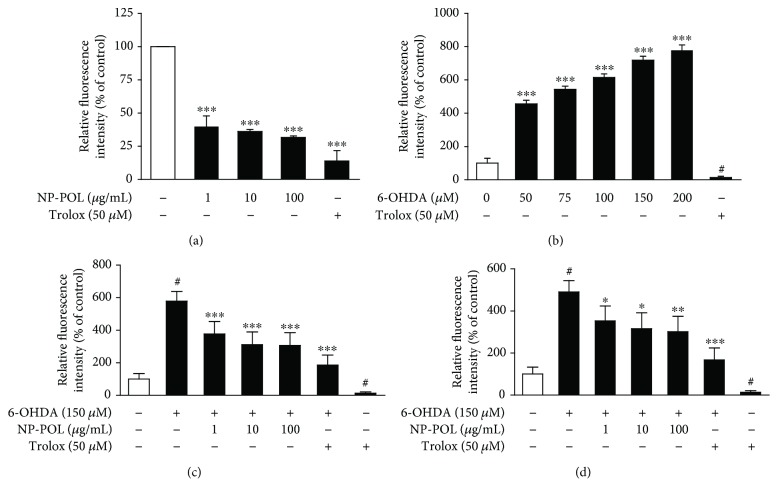
The effect of the NP-POL peptide on intracellular ROS generation induced by 6-OHDA. (a) NP-POL significantly reduced intracellular ROS levels in PC12 cells. (b) 6-OHDA induced an exponential increase in intracellular ROS in exposed cells. Both 1 h preincubation (c) with selected doses of NP-POL before 24 h of 6-OHDA exposure and 24 h of coincubation with NP-POL and 6-OHDA (d) resulted in a significant decrease in intracellular ROS levels. Data are expressed as mean ± SD (*n* = 3). Results were considered significant at ^#^ *p* < 0.001 versus untreated cells and ^∗^ *p* < 0.05, ^∗∗^ *p* < 0.01, and ^∗∗∗^ *p* < 0.001 versus 6-OHDA.

Therefore, investigation of the effect of NP-POL peptide on ROS generation was the next goal of our study. Firstly, using the DPPH method, FRAP method, and Fe^2+^ scavenging method, we revealed no antioxidant capacity of NP-POL itself (see supplementary data). Next, the effect of NP-POL on 6-OHDA-induced ROS generation was examined. PC12 cells treated with NP-POL showed a significant decrease in DCF fluorescence intensity in a dose-dependent manner (39.8% ± 8%, 36.2% ± 1%, and 31.7% ± 0.9% for 1 *μ*g/ml, 10 *μ*g/ml, and 100 *μ*g/ml of NP-POL, respectively; all data was expressed as % of control) (Figure 5(a)). In comparison, in 6-OHDA-treated cells, a significant increase of DCF fluorescence intensity was observed (Figure 5(b)). This effect was also dose-dependent, while a sevenfold increase (719% ± 22.9% versus control 100%) was observed at the dose of 150 *μ*M. However, 1 h preincubation and coincubation of NP-POL with 6-OHDA (150 *μ*M) significantly reduced ROS generation when compared to the 6-OHDA applied alone (1 h preincubation with NP-POL: 6-OHDA alone: 578% ± 60.3%, NP-POL 1 *μ*g/ml and 6-OHDA: 377% ± 76.8%, NP-POL 10 *μ*g/ml and 6-OHDA: 312.5% ± 77.3%, and NP-POL 100 *μ*g/ml and 6-OHDA: 307% ± 78.2% (Figure 5(c)); simultaneous application: 6-OHDA alone: 490.95 ± 54%, NP-POL 1 *μ*g/ml + 6-OHDA: 353% ± 70.9%, NP-POL 10 *μ*g/ml + 6-OHDA 315% ± 75.6%, and NP-POL 100 *μ*g/ml + 6-OHDA 301.6% ± 73.3%) (Figure 5(d)). The same inhibitory effect of NP-POL was observed when H_2_O_2_ was used as a source of ROS (data not shown).

Previous studies have investigated the inhibitory effects of PRP on oxidative stress and ROS generation. It was confirmed that PRP effectively and specifically reduces the generation of ROS, protein, and lipid oxidation, regulates glutathione metabolism, and improves antioxidant system activity [25, 39]. These findings suggest that PRP maintenance towards intracellular antioxidant homeostasis is due to the biological capacities of its constituent peptides. One of them is the constituent NP-POL peptide.

#### 3.3.4. NP-POL Nonapeptide Regulates Activity of ERK 1/2 MAP Kinases

ERK 1/2 kinase, one of the most well-characterized members of the mitogen-activated protein (MAP) kinase family, regulates a range of processes from metabolism and inflammation to cell death and survival. In the nervous system, ERK 1/2 regulates synaptic plasticity, brain development, and repair, as well as cellular response to stress factors [40, 41]. 6-OHDA-induced oxidative stress mediates the cellular response of survival and apoptosis involving, besides p38 MAPK and JNK kinases, downstream kinases of ERK 1/2 signaling promoting cell survival [42]. A number of reports indicate that transient phosphorylation of ERK 1/2 kinases may protect against 6-OHDA-induced cytotoxicity in neuronal cells via the PKA/Bcl2-dependent pathway [41, 42]. In our present work, we showed that PRP possesses neuroprotective activity in its ability to activate the cGMP/ERK 1/2 signaling pathway in PC12 cells [26]. Therefore, it was important to check whether the neuroprotective effect of NP-POL on 6-OHDA-treated PC12 cells may be related to its influence on ERK 1/2 activation.

**Figure 6:**
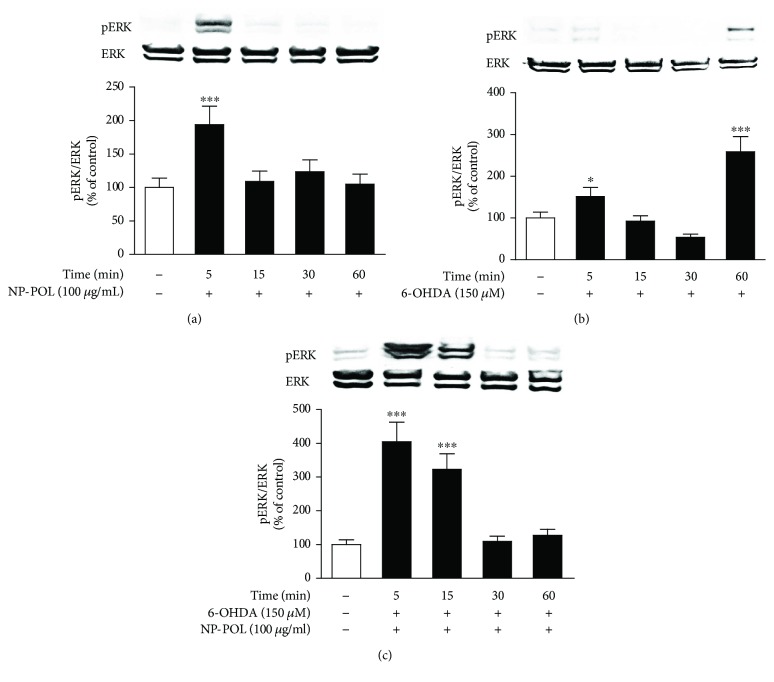
The effect of NP-POL peptide on ERK 1/2 kinase activity. (a) NP-POL transiently enhanced ERK 1/2 activation at the selected dose of 100 *μ*g/ml after 5 min of incubation. (b) Stable 6-OHDA-enhanced ERK 1/2 activation after 60 min of incubation. (c) Coincubation of NP-POL with 6-OHDA resulted in the transient activation of ERK 1/2 after 5 and 15 min. Data are presented as mean ± SD (*n* = 3). Results were considered significant at ^∗^ *p* < 0.05 and ^∗∗∗^ *p* < 0.001 versus untreated cells.

It was shown that the NP-POL nonapeptide at 100 *μ*g/ml induced the transient phosphorylation of ERK 1/2 in PC12 cells after 5 min, compared to the control group (Figure 6(a)). PC12 cells treated with 6-OHDA at 150 *μ*M induce sustained ERK 1/2 phosphorylation for 60 min (Figure 6(b)). However, the sustained phosphorylation of ERK 1/2 induced by 6-OHDA (150 *μ*M) was reduced by coincubation with 100 *μ*g/ml of NP-POL nonapeptide for 60 min with a simultaneous induction of transient phosphorylation of ERK 1/2 at 5–15 min (Figure 6(c)). Kulich and Chu [43, 44] found that catalase was capable of attenuating 6-OHDA-mediated sustained ERK phosphorylation. Therefore, we can speculate that the NP-POL nonapeptide could inhibit 6-OHDA-mediated sustained ERK 1/2 activation and toxicity by the effect of NP-POL on catalase activation.

## 4. Conclusions

In the present work, we demonstrated for the first time a method of isolation of the new PRP component, NP-POL nonapeptide with the RPKHPIKHQ sequence. NP-POL was isolated from PRP by Bio-Gel P2 molecular sieve beads using 50 mM EDTA. The NP-POL nonapeptide showed a neuroprotective effect on PC12 cells treated with neurotoxic 6-OHDA (6-hydroxydopamine) exerting a beneficial effect on the regulation of cell survival and the inhibition of ROS overproduction released during 6-OHDA metabolism, probably by its ability to activate the antioxidant system. Additionally, this effect was connected with transient ERK 1/2 kinase activation. Thus, these results suggest that the NP-POL nonapeptide would likely be a promising agent in the treatment of neurodegenerative diseases, such as Parkinson's disease.

## Data Availability

The data used to support the findings of this study are available from co-corresponding authors upon request.

## Supplementary Materials

Supplementary Materials Figure S1. Elution profile of peptide fraction “c1” separated by RP-HPLC. Fraction “c1” previously isolated by Bio-Gel P2 was dissolved in 0.1% TFA and loaded on a Nucleosil 100 C-18 column. The proteins were eluted with an acetonitrile linear gradient (0%–60% for 80 min) containing 0.1% TFA at a flow rate of 1 ml/min. Figure S2. SDS-PAGE analysis of peptide fraction “c” obtained by the molecular sieve beads Bio-Gel P2. The samples (10 mg) were loaded on to gel slabs (12%). The gel slabs were cast 24 h ahead of electrophoresis and stained with Coomassie Brilliant Blue R 250 for proteins. NP POL localization: line 2, MWM—molecular size marker. Table S1. Comparison of the amino acid sequence of NP POL nonapeptide with a sequence of homologous proteins. The search for homologous proteins based on UniProt protein sequences was carried out using the FastA3 software package. NP POL nonapeptide showed 100% homology to the 16- to 24-amino acid fragment of the sheep and beef alpha s1 casein precursor and 88.88% identity with the alpha s1 casein. Table S2. Antioxidant capacity of the NP-POL peptide measured in tests conducted *in vitro*. The antioxidant activity of the NP POL nonapeptide (0.1 *μ*g/ml–100 *μ*g/ml) was assessed by DPPH, FRAP, and Fe(II) ion chelation methods (for details see Sections 2.6, 2.7, and 2.8). All data are expressed as mean values ± SD, *n* = 3.
