# Supplementary material for: Isolation and Characterization of NP-POL Nonapeptide for Possible Therapeutic Use in Parkinson's Disease
Source: Oxid Med Cell Longev. 2018 Jul 18;2018:3760124. doi: 10.1155/2018/3760124 (PMC6079319; doi:10.1155/2018/3760124)
Supplement: Supplementary Materials — Figure S1. Elution profile of peptide fraction “c1” separated by RP-HPLC. Fraction “c1” previously isolated by Bio-Gel P2 was dissolved in 0.1% TFA and loaded on a Nucleosil 100 C-18 column. The proteins were eluted with an acetonitrile linear gradient (0%–60% for 80 min) containing 0.1% TFA at a flow rate of 1 ml/min. Figure S2. SDS-PAGE analysis of peptide fraction “c” obtained by the molecular sieve beads Bio-Gel P2. The samples (10 mg) were loaded on to gel slabs (12%). The gel slabs were cast 24 h ahead of electrophoresis and stained with Coomassie Brilliant Blue R 250 for proteins. NP POL localization: line 2, MWM—molecular size marker. Table S1. Comparison of the amino acid sequence of NP POL nonapeptide with a sequence of homologous proteins. The search for homologous proteins based on UniProt protein sequences was carried out using the FastA3 software package. NP POL nonapeptide showed 100% homology to the 16- to 24-amino acid fragment of the sheep and beef alpha s1 casein precursor and 88.88% identity with the alpha s1 casein. Table S2. Antioxidant capacity of the NP-POL peptide measured in tests conducted in vitro. The antioxidant activity of the NP POL nonapeptide (0.1 μg/ml–100 μg/ml) was assessed by DPPH, FRAP, and Fe(II) ion chelation methods (for details see Sections 2.6, 2.7, and 2.8). All data are expressed as mean values ± SD, n = 3. [file 3760124.f1.zip › Table S1.doc]

| NPPOL | 1 9  RPKHPIKHQ |
| --- | --- |
|  | 10 20 30 40 |
| CASA1_SHEEP Alpha-S1-casein precursor (206 aa) | MKLLILTCLVAVALARPKHPIKHQGLSSEVLNENLLRFVV |
| CASA1_SHEEP Alpha-S1-casein precursor (214 aa) | MKLLILTCLVAVALARPKHPIKHQGLSSEVLNENLLRFVV |
| CASA1_BOVIN Alpha-S1-casein precursor (214 aa) | MKLLILTCLVAVALARPKHPIKHQGLPQEVLNENLLRFFV |
| Q8MIH2_CAPHI Alpha s1 casein (115 aa) | MKLLILTCLVAVALARPKHPINHQGLSPEVLNENLLRFVV |
| Q69EZ6_CAPHI CSCN1 (213 aa) | MKLLILTCLVAVALARPKHPINHQGLSPEVPNENLLRFVV |
| Q8MIH4_CAPHI Alpha s1 casein (214 aa) | MKLLILTCLVAVALARPKHPINHQGLSPEVLNENLLRFVV |
